# Relationship between ABO blood group antigens and Rh factor with breast cancer: A systematic review and meta-analysis

**DOI:** 10.18632/oncotarget.28718

**Published:** 2025-05-09

**Authors:** Rahaf Alchazal, Khaled J. Zaitoun, Mohammad Al-Qudah, Ghena Zaitoun, Amira M. Taha, Othman Saleh, Mohammad Alqudah, Mohammad Abuawwad, Mohammad Taha, Abdullah Yousef Aldalati

**Affiliations:** ^1^Faculty of Medicine, Yarmouk University, Irbid, Jordan; ^2^Department of Neurosurgery, Johns Hopkins University School of Medicine, Baltimore, MD 21205, USA; ^3^Faculty of Medicine, Jordan University of Science and Technology, Irbid, Jordan; ^4^Faculty of Medicine, Hashemite University, Amman, Jordan; ^5^Department of Biology, Edmonds College, Lynnwood, WA 98036, USA; ^6^Faculty of Medicine, Fayoum University, Egypt; ^7^Faculty of Medicine, Cairo University Kasr Alainy, Egypt; ^*^These authors contributed equally to this work

**Keywords:** breast cancer, cancer risk factors, blood group antigens, tumor

## Abstract

Background: Breast cancer is a type of cancer that can affect both males and females, but it is widespread among women. Blood types may be associated with breast cancer, as many studies have reported on this relationship but rarely described it. The primary objective of our research is to summarize and analyze the available evidence to produce comprehensive and accurate information that can be used to make evidence-based decisions.

Methods: Researchers searched for studies on breast cancer patients and ABO blood groups across four major databases: PubMed, Scopus, Web of Science, and Google. The outcomes of the studies were presented as a relative risk and odds ratio with a 95% confidence interval.

Results: Twenty-nine case-control studies with 13029 breast cancer patients. Blood type A was the most common blood type among patients. For blood type A, there was an association with breast cancer (OR = 1.18, 95% CI: 1.03–1.36). Blood types B, AB, and Rh factor showed no significant association with breast cancer (OR = 0.97, 95% CI: 0.86–1.11, OR = 1.05, 95% CI: 0.89–1.25, and OR = 1.14, 95% CI: 0.81–1.60 respectively) in compare to blood type A.

Conclusions: This study highlights the potential of blood type A as a risk factor for breast cancer compared to blood type O. This relationship was insignificant for blood types B, AB, or Rh. Further studies are needed to understand the mechanisms behind the blood type and breast cancer correlation.

## INTRODUCTION

Breast cancer is the most prevalent form of cancer among females, and the second-leading cause of cancer mortality [1, 2]. Breast cancer mainly occurs in middle-aged and older women [3]. The cause of this neoplasm is unclear, but available epidemiological data point to three major contributors that may be important in breast cancer: environmental, genetic, and hormonal factors [4]. Many risk factors are associated with the development of breast cancer; ABO blood groups are rarely considered as a risk factor for breast cancer. However, some studies reported a causal relationship between certain blood groups and increased susceptibility to breast cancer and not a cause of breast cancer [5].

The relationship between blood type and disease has been studied for many years, uncovering numerous associations between specific blood types and an increased risk of various illnesses. The correlation between the ABO blood group system and cancer received notable attention in the mid-20th century [6]. Interest in this topic has increased since 1921 [7] when correlations between blood groups of the ABO system and pancreatic cancer were documented [8–10].

The ABO blood type system describes the expression of the ABH antigens found on both erythrocytes and normal tissue throughout the body such as the lungs, GI tract, breast, uterine cervix, mouth, prostate, and bladder [9]. Blood group antigens have specific functions, including the capacity to promote blood clotting and blood vessel development [11]. Additionally, antigens act as ligands for selection and aid in cell adhesion [12]. These biological characteristics might contribute to cancer’s growth [11–13]. Due to these characteristics, cells resist programmed death [11] and migrate to adjacent tissues [12].

It is well known that these antigens’ expression changes during the process of cell growth and differentiation, and throughout the body’s aging process. These changes in expression have a major impact on cell-related diseases such as cancer [5].

ABO glycoproteins are misexpressed in malignant tissue, according to histological comparisons of normal and neoplastic tissue [14–19]. Loss of A and B antigens in malignant tissue and oncological antigens are obtained and act as ligands for selectins [19]. Normal antigens are lost; tumor antigens are acquired, leading to metastasis [20]. These biological characteristics, antigen changes, and missing may play a role in increased susceptibility to growth and metastatic cancer.

Compared to blood type O, people with blood type A have a higher incidence of malignancies in the stomach, ovaries, salivary glands, cervix, uterus, and colon/rectum [6, 20, 21] However, several studies have emerged evaluating the association between blood type and breast cancer, yet the available evidence is inconsistent and does not provide conclusive results to support or refute the proposed hypotheses [21–23]. Studies still confirm this relationship, although the mechanism behind it is still not fully understood.

The primary objective of our study is to summarize and analyze available evidence about the relationship between the ABO blood group system and breast cancer. We aim to produce a comprehensive report that provides accurate and comprehensive information that can be used to make evidence-based decisions.

## RESULTS

### Database search results

**Figure 1:**
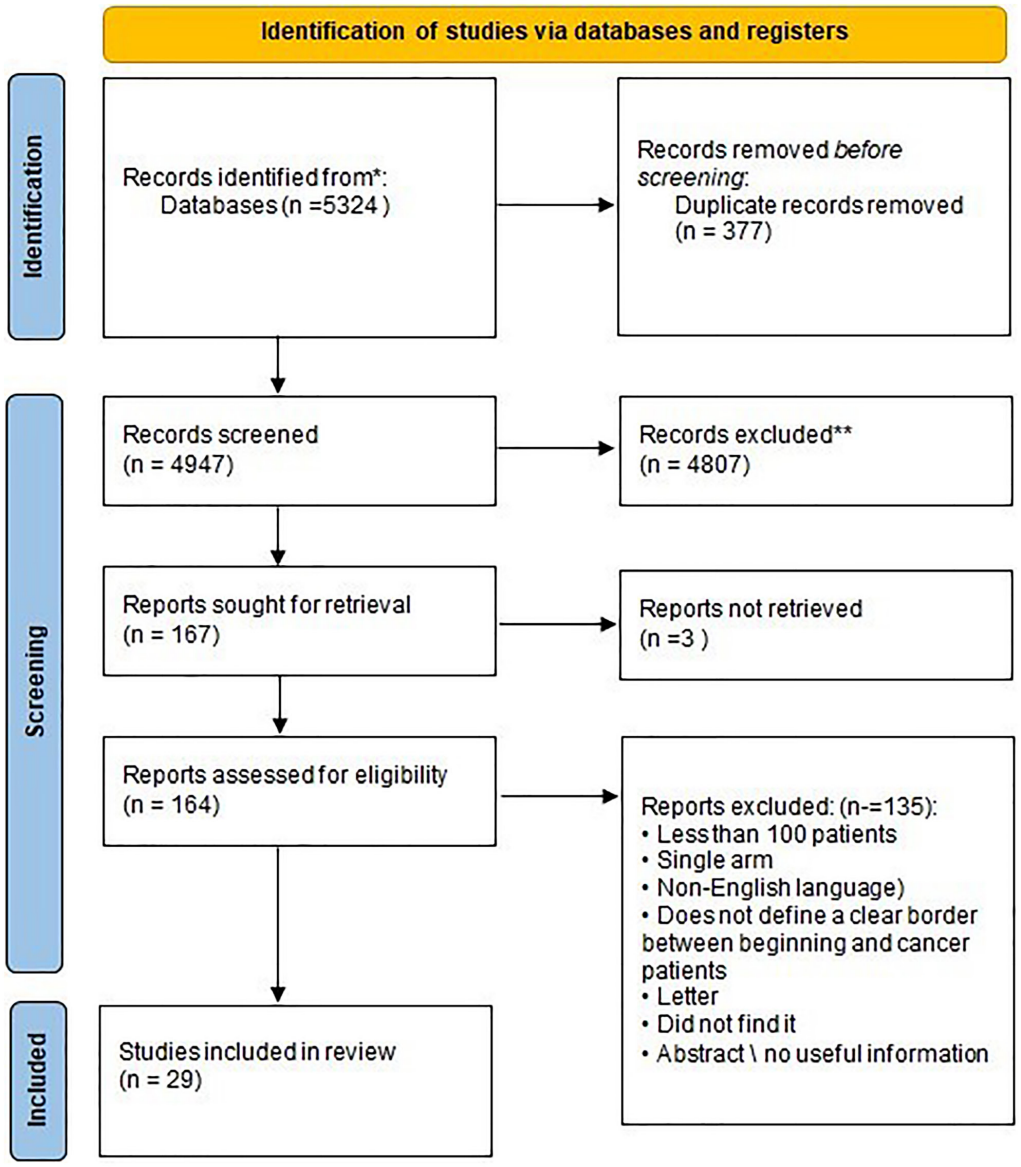
PRISMA flow chart of the included studies.

The details of the database search and screening processes are illustrated in the PRISMA diagram, shown in Figure 1. Following the retrieval of studies from the initial four databases (WOS = 1184, PubMed = 160, Scopus = 3012 and Google Scholar = 968), 377 duplicated records were found and removed through EndNote software and. Then, the titles and abstracts of 4947 records were screened against our eligibility criteria, yielding 164 records that warranted further full-text screening. Finally, 29 studies were included in the qualitative and quantitative synthesis of our review.

### Characteristics of the included studies

Twenty-nine studies included 13029 patients with breast cancer and 717848 controls. Among the breast cancer patients, blood group A was the most reported, followed by O, B and AB. All included studies were case-control (Table 1) distribution of blood types and rh factors in case and control groups were shown in (Table 2) and the classification of breast cancer cases by histology type and receptor status was shown in (Tables 3 and 4).

**Table 1:** Baseline characteristics of the included studies

Authors	Year	Study title	Location	Source of control	Study design	Case age, mean(SD)	Female control, (%)	Female case, (%)
Abbasi et al. [24]	2009	Risk Factors for Breast Cancer in Iranian Women: A Case-Control Study	Iran	Hospital	Case Control		100%	100%
Abobaker et al. [25]	2014	Incidence of Breast Cancer in a Primary Hospital in Relation to ABO Blood Groups System	Penang, Malaysia	Hospital	Case Control		100%	32%
Akammu et al. [4]	2002	The association between cancer of the breast and the abo and rhesus d antigen phenotypes in lagos, nigeria: a case-control study	Lagos, Nigeria	Hospital	Case Control		99%	
Al-Ganimi et al. [26]	2020	The Relation between the Blood Groups, Rhesus Factor, and Breast Cancer in the Holy Karbala Governorate	Karbala, Iraq	Hospital	Case Control	43.08 ±1.37		
Aly et al. [27]	2014	Association of ABO Blood Group and Risk of Breast Cancer	Egypt	Hospital	Case Control	62.0 ± 4	100%	100%
Anderson et al. [28]	1984	Blood Type A and Familial Breast Cancer	Texas, US	Mixed	Case Control			
Aird, et al. [29]	1954	The blood groups in relation to peptic ulceration and carcinoma of colon, rectum, breast, and bronchus	England	Mixed	Case Control		100%	
Bhartiaya et al. [30]	2015	Association of ABO Blood Group in Breast Cancer	India	Hospital	Case Control	Median (Range); 55 (25–87)	100%	100%
Bothou et al. [31]	2019	Blood groups type linked to breast cancer in a Greek cohort of women - a case control study	Athens, Greece	Hospital	Case Control		100%	100%
Costantini et al. [32]	1990	Role of Blood Groups as Prognostic Factors in Primary Breast Cancer	Italy, Genoa	Hospital	Case Control			
DIXIT et al. [33]	2020	Epidemiological study of breast cancer patients and their association with abo blood group	India	Hospital	Case Control		100%	100%
Dogan et al. [34]	2022	The frequency and prognostic significance of ABO/Rh blood groups in male breast cancer patients A multicenter study	Turkey	Social	Case Control		100% Male	100% Male
Flavargani et al. [35]	2012	Study of the association between blood types and breast cancer among Isfahanian women with breast cancer	Iran	Social	Case Control		100%	100%
Kumar et al. [36]	2015	Incidence of Breast Cancer and ABO Blood group: A Hospital Based Study	India	Social	Case Control		100%	100%
Mehdi et al. [37]	2008	Relationship between ABO blood group and breast cancer at AL-Nassyria city/Iraq	Iraq	Social	Case Control			
Mjali et al. [38]	2019	Association between Female Breast Cancer and Different ABO Blood Groups & Rh Factor in the Sulaymaniyah Province of Iraqi Kurdistan	Iraq	Social	Case Control	47.37 ± 9.7	100%	100%
Mohammad et al. [39]	2016	The Association and Relation of ABO Blood Group and Secretor status with the Breast Cancer	Iraq	Social	Case Control		100%	100%
Newell et al. [40]	1974	ABO Blood Groups and Cancer	United States	Social	Case Control		100%	100%
Ronco et al. [41]	2009	Rh factor, family history and risk of breast cancer: A case–control study in Uruguay	Uruguay	Social	Case Control		100%	100%
Stamatakos et al. [42]	2009	Breast cancer incidence in Greek women in relation to ABO blood groups and Rh factor	Greece	Hospital	Case Control	55.31	100%	100%
Zaki et al. [43]	2013	The Association and Relation of ABO Blood Group with the Breast Cancer in Kirkuk Governorate	Kirkuk, Iraq	Hospital	Case Control		100%	100%
Zouine et al. [44]	2016	ABO blood groups in relation to breast carcinoma incidence and associated prognostic factors in Moroccan women	Morocco	Social	Case Control	62.1 ± 12.8	100%	
Goldenberg et al. [45]	1958	Breast carcinoma and abo blood groups	United States	Social	Double arm Retrospective			
Sujatha et al. [46]	2016	Association of ABO blood group and risk of female breast cancer-A retrospective study	India	Hospital	Double arm	45.6 ± 11.6	100%	
Joudaki et al. [47]	2022	Study of the Relationship between ABO Blood Group Types and Breast Cancer and Cervix Cancer in Khuzestan Province, Iran	Iran	Social	A retrospective study.			
Alhusseini et al. [48]	2016	Study of blood groups antigens frequency for ductal breast carcinoma patients in some Iraqi women	Iraq	Social	Case Control		100%	100%
Urun et al. [49]	2012	ABO and Rh blood groups frequency in women with HER2 positive breast cancer	Turkey	Social	Case Control	Median (Range); 47 (20–80)	100%	100%
Ray et al. [50]	1980	Blood Groups and Cancer in India	India	Social	Case Control			
Hartmann et al. [51]	1964	Abo blood-groups and cancer	Norway	Mixed	Case Control			

**Table 2:** Distribution of blood types and Rh factor in case and control groups

First author (Year)	Case				Control				Total	Case	Control
	A	B	AB	O	A	B	AB	O			
Abbasi [24] (2009)	27	14	6	103	43	35	16	53	297	150	147
Abobaker [25] (2014)	21	17	13	19	30	35	16	59	210	70	140
Aird [29] (1954)	417	102	29	469	14956	3351	1108	17611	38043	1017	37026
Akammu [4] (2002)	28	22	3	54	616	465	71	1213	2472	107	2365
Al-Ganimi [26] (2020)	135	128	56	220	88	69	31	125	852	539	313
Alhusseini [48] (2016)	25	10	22	43	27	24	11	38	200	100	100
Aly [27] (2014)	85	28	12	35	36	17	9	30	252	160	92
Anderson [28] (1984)	1061	257	94	1137	7505	2042	668	8881	21645	2549	19096
Bhartiaya [30] (2015)	53	41	25	45	30	86	16	88	384	164	220
Bothou [31] (2019)	125	11	12	54	44	19	10	66	341	202	139
Costantini [32] (1990)	130	47	12	126	8440	2431	847	9126	21159	315	20844
Dixit [33] (2020)	42	78	12	55	29	80	15	85	396	187	209
Dogan [34] (2022)	52	26	24	35	51386	18590	9624	42560	122297	137	122160
Flavargani [35] (2012)	50	41	9	73	119	87	17	153	549	173	376
Goldenberg [45] (1958)	413	111	29	447	30590	8881	3264	33170	76915	1000	75915
Hartmann [51] (1964)	830	109	56	605	15139	2703	1173	12476	33091	1600	31491
Joudaki [47] (2022)	24	26	9	50	8378	7480	2095	11969	30031	109	29922
Kumar [36] (2015)	85	140	29	146	56	75	25	44	600	400	200
Mehdi [37] (1991)	120	20	20	40	72	77	23	107	479	200	279
Mjali [38] (2019)	136	70	39	255	208	79	40	173	1000	500	500
Mohammad [39] (2016)	57	10	4	40	21	3	1	25	161	111	50
Newell [40] (1974)	125	75	22	215	164	94	28	264	987	437	550
Ray [50] (1980)	273	558	79	388	2114	3002	719	2986	10119	1298	8821
Ronco [41] (2009)	93	5	17	137	166	34	42	307	801	252	549
Stamatakos [42] (2009)	79	23	9	55	130	29	17	124	466	166	300
Sujatha [46] (2016)	39	26	4	31	39	26	4	31	200	100	100
Urun [49] (2012)	145	42	21	86	9925	3610	1786	7500	23115	294	22821
Zaki [43] (2013)	160	24	21	45	88	62	42	108	550	250	300
Zouine [44] (2016)	131	88	16	207	117048	52035	14946	158794	343265	442	342823
Total	4961	2149	704	5215	267487	105521	36664	308166	730877	13029	717848

**Table 3:** Classification of breast cancer cases by histology type and receptor status

Authors	Year	Histology type			ER status		
		Invasive ductal carcinoma	Other types	Unknown	+	–	Unknown
Abbasi et al. [24]	2009				40	92	18
Akammu et al. [4]	2002	93	14				
Al-Ganimi et al. [26]	2020				90	70	
Costantini et al. [32]	1990	315			144	165	5
DIXIT et al. [33]	2020	86	101		27	72	
Dogan et al. [34]	2022	110	20	7	120	7	10
Flavargani et al. [35]	2012	147	26				
Mjali et al. [38]	2019	459	41		379	121	
Ronco et al. [41]	2009	203	25	24			
Stamatakos et al. [42]	2009	119	47		106	15	
Zouine et al. [44]	2016	391	61		171	78	193
Joudaki et al. [47]	2022	80	24	5	29	10	
Alhusseini et al. [48]	2016	100					
Urun et al. [49]	2012	268	26		204	190	6

**Table 4:** PR status and HER2 overexpression

Authors	Year	PR status			HER2 overexpression		
		+	−	Unknown	+	–	Unknown
Al-Ganimi et al	2020	120	40	90			
DIXIT et al	2020	28	70		67	32	
Dogan et al	2022	95	31	11	27	97	13
Mjali et al	2019	360	140				
Stamatakos et al	2009	101	20				
Zouine et al	2016	125	124	193	49	190	203
Joudaki et al	2022	22	17		12	18	
Urun et al	2012	236	153	12			

### Quality assessment

**Figure 2:**
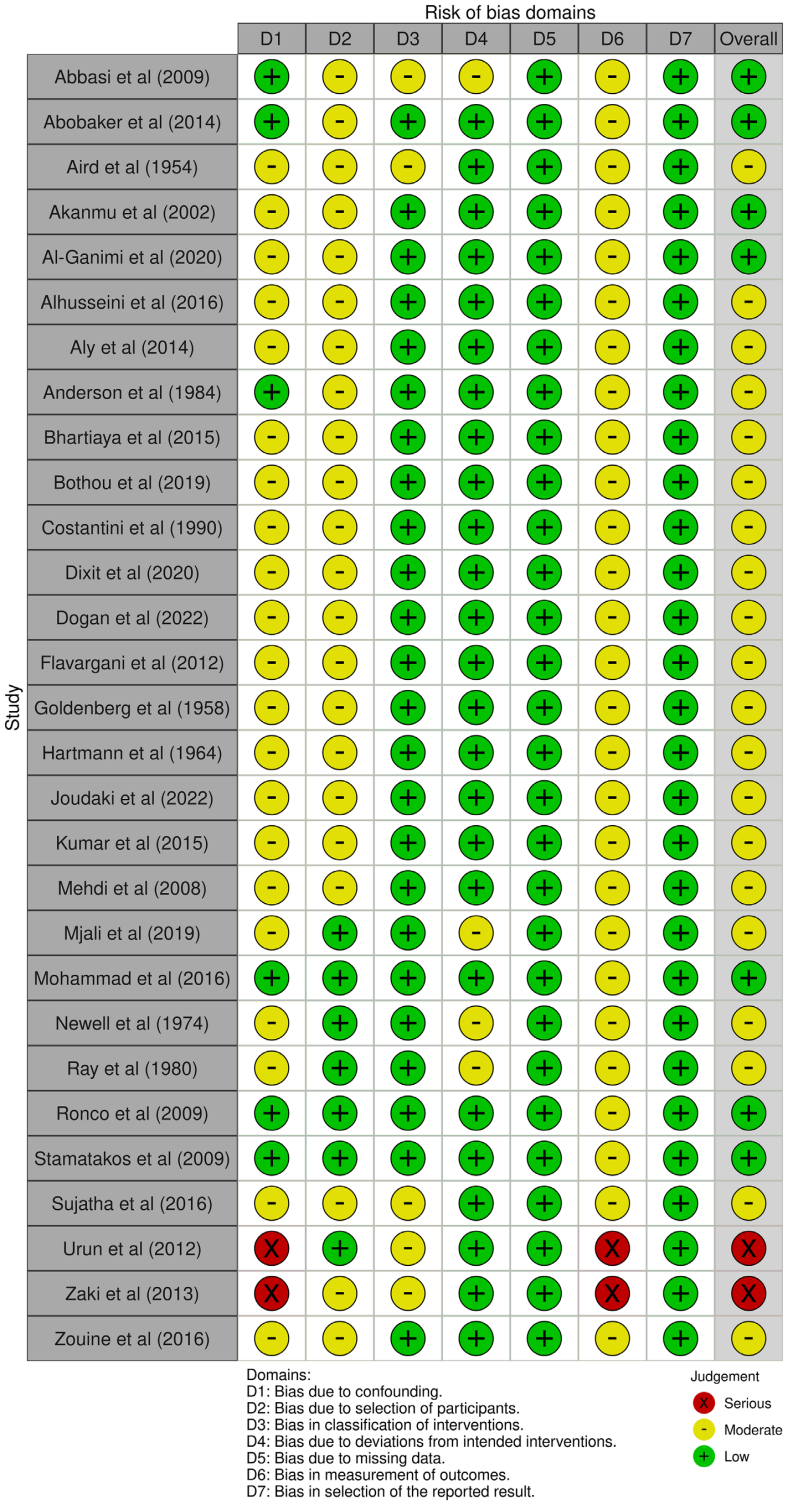
Bar plot showing the risk of bias assessment for the included studies.

**Figure 3:**
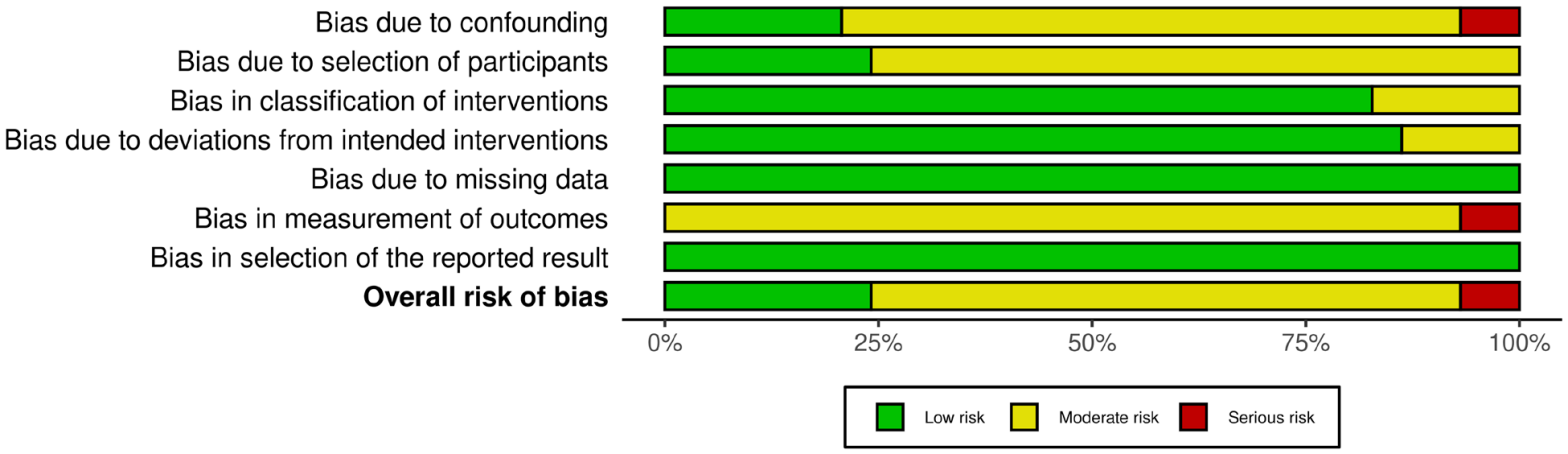
Traffic light plot illustrating the risk of bias assessment for the included studies.

The assessment of bias risk revealed a low risk in seven, a moderate risk in twenty studies, and a high risk in two of them. Nearly all the studies showed a high risk in the confounding domain. These findings are graphically demonstrated using traffic light and bar plots in Figures 2 and 3.

### Associations between ABO blood groups, Rh factor, and breast cancer risk

**Figure 4:**
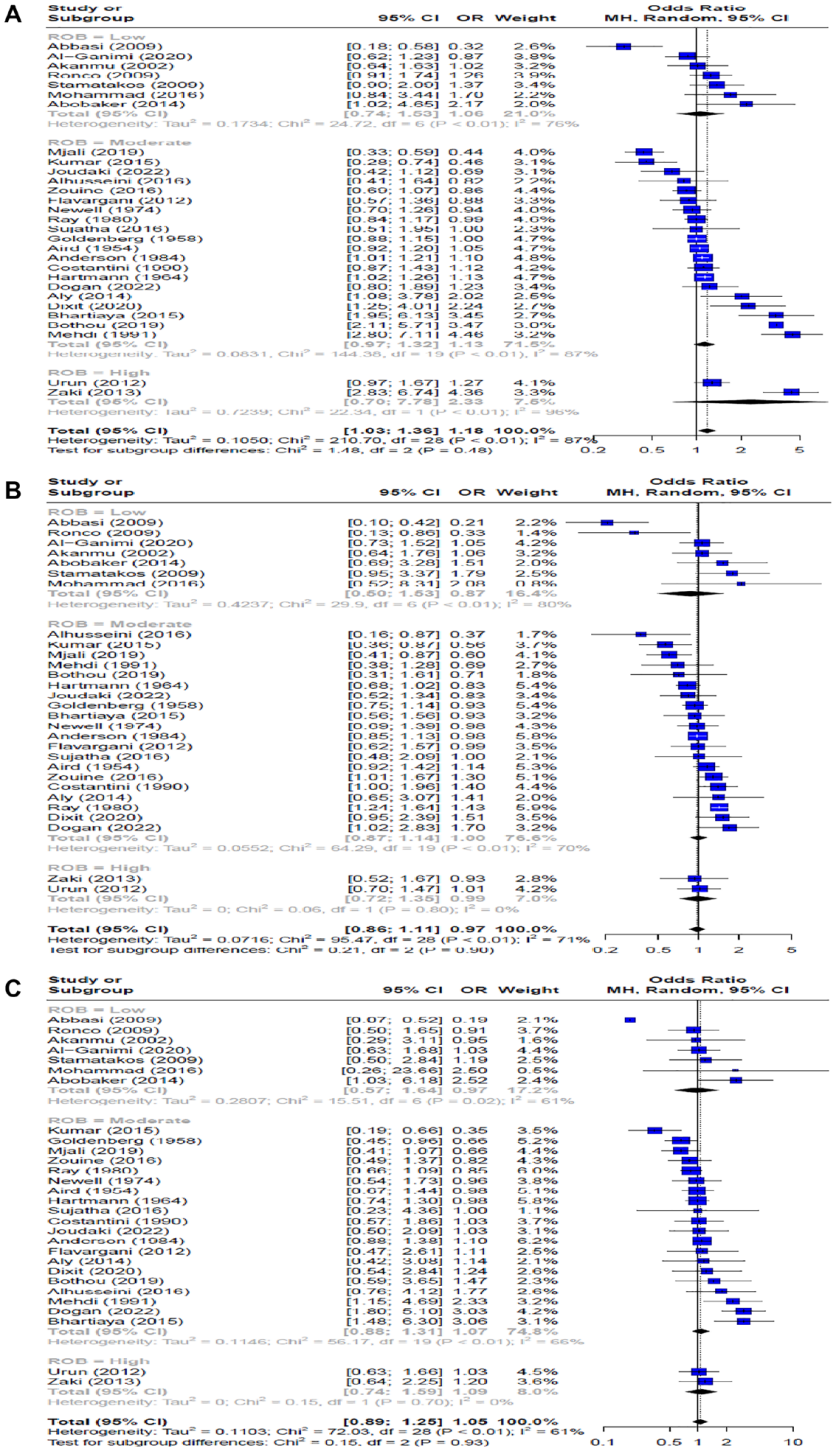
Forest plot of the odds ratios for breast cancer risk comparing blood groups. (**A**) A to O, (**B**) B to O, (**C**) AB to O.

**Figure 5:**
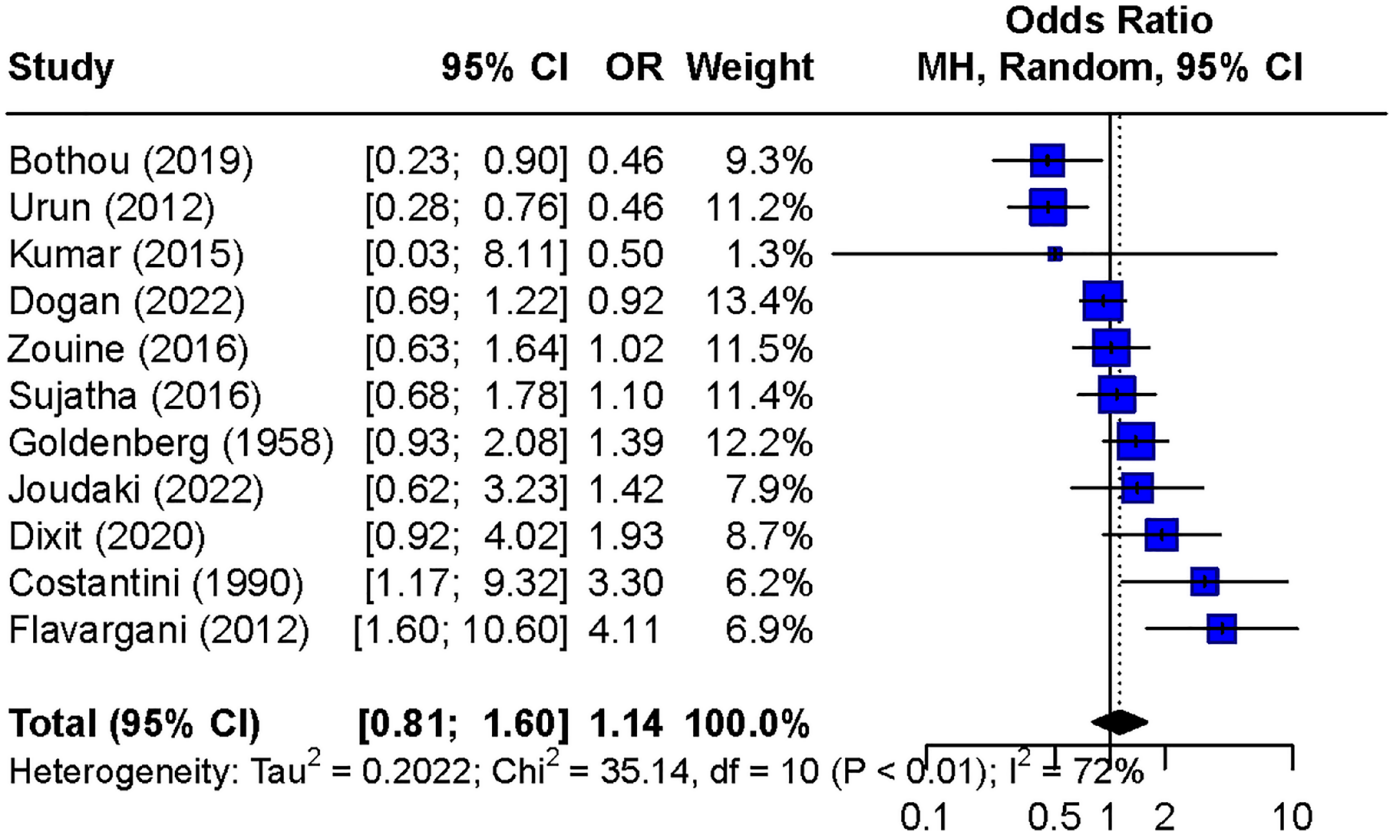
Forest plot of the odds ratios for breast cancer risk comparing Rh positive to Rh negative.

For blood type A, there was a statistically significant association with breast cancer (OR = 1.18, 95% CI: 1.03–1.36) (Figure 4A), demonstrating high heterogeneity (I^2^ = 87%). Blood types B (Figure 4B) and AB (Figure 4C) showed no significant association with breast cancer (OR = 0.97, 95% CI: 0.86–1.11 and OR = 1.05, 95% CI: 0.89–1.25, respectively), both with substantial heterogeneity (I^2^ = 71% for B and I^2^ = 61% for AB). In the analysis of the Rh factor, employing the random effects model on 11 studies, no significant association was found between Rh factor and breast cancer risk (OR = 1.14, 95% CI: 0.81–1.60), with substantial heterogeneity (I^2^ = 72%). (Figure 5).

**Figure 6:**
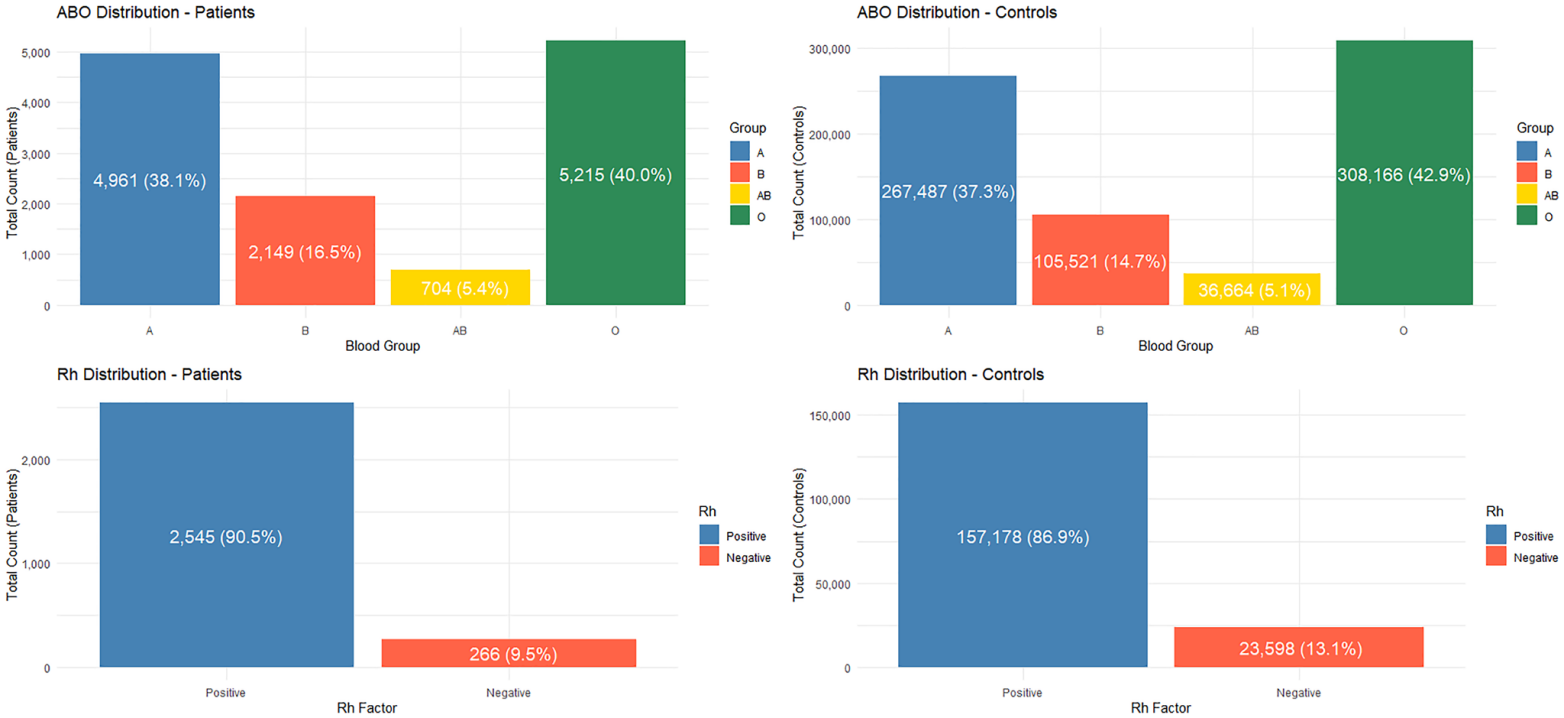
Bar plot showing patient counts for ABO and Rh groups, with each bar labeled by its count and corresponding percentage.

Figure 6 illustrates the distribution of patient counts across the various blood groups and Rh factors.

A subgroup analysis comparing studies conducted in Asia with those from non-Asian regions showed no statistically significant difference in the association between blood group A and breast cancer risk (*P* = 0.74). The pooled OR in the non-Asian group was statistically significant (OR = 1.13, 95% CI: 1.01–1.26), whereas the OR in the Asian group was not (OR = 1.19, 95% CI: 0.86–1.66). (Supplementary Figure 1).

### Assessing publication bias

**Figure 7:**
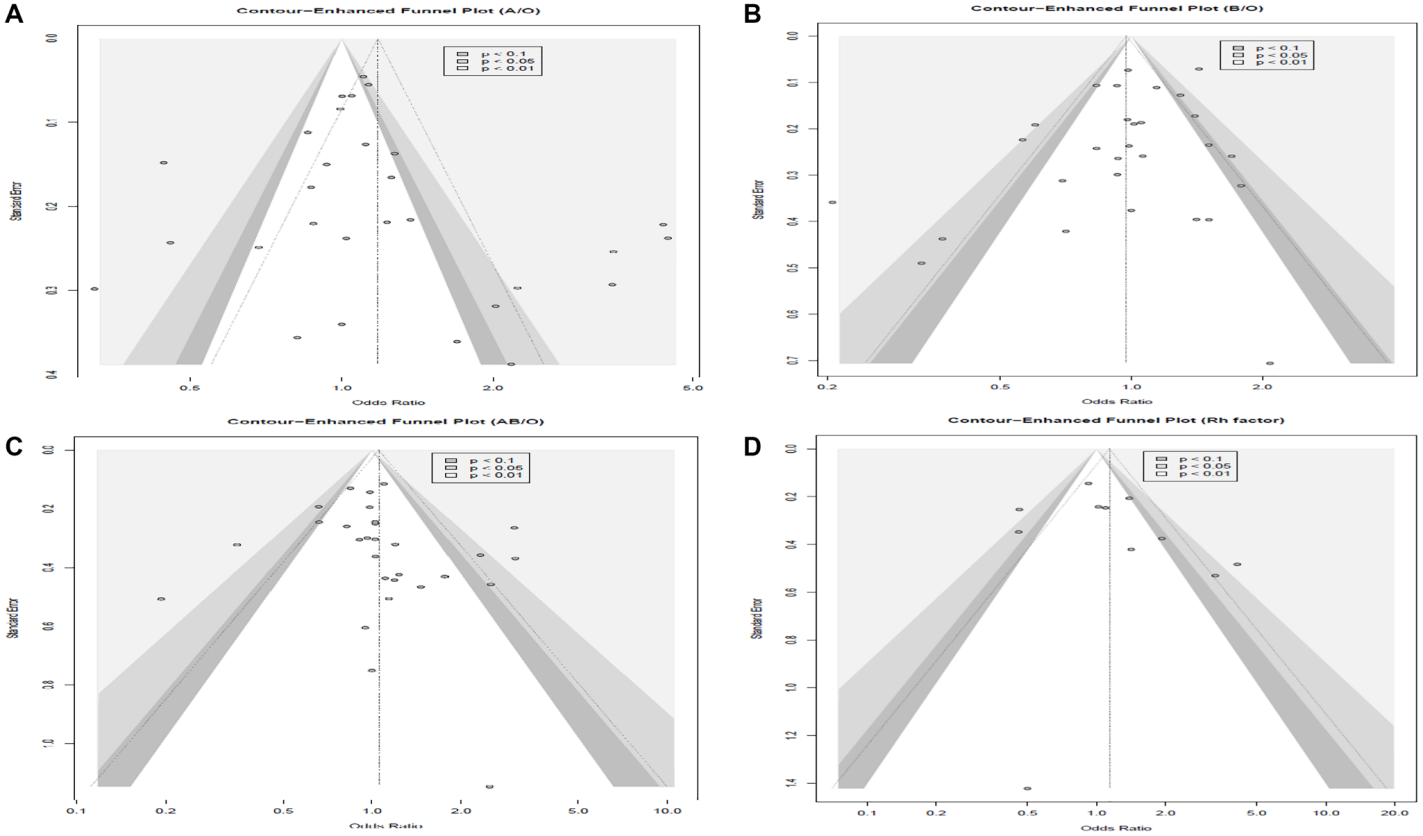
Funnel plots for blood group comparisons. (**A**) A to O, (**B**) B to O, (**C**) AB to O, (**D**) Rh positive to Rh negative.

A combination of visual and statistical methods was used to evaluate publication bias. Despite the Egger’s test outcomes (Supplementary Table 1) indicating no significant publication bias across the blood group comparisons (A/O, B/O, AB/O) (Figure 7A–7C) and Rh factor, visual inspection of the funnel plots for A/O, B/O, and AB/O comparisons suggested potential asymmetry. Conversely, for the Rh factor, both visual inspection and Egger’s test suggested minimal publication bias. (Figure 7D).

## DISCUSSION

This meta-analysis provides evidence for the possible associations between blood types and breast cancer risk. Our study, which included data from 29 studies, found a significant association between blood type A and an elevated risk of breast cancer when compared to blood type O. This relationship was statistically significant, with an odds ratio (OR) of 1.18 and a 95% CI of 1.03–1.36. However, it is noteworthy to emphasize the high heterogeneity identified in this comparison (I^2^ = 87%), which indicates significant variability among the included studies. Contrary to blood type A, our analysis did not find any significant associations between blood types B and AB and breast cancer risk when compared to blood type O. Furthermore, our analysis of the Rh factor, encompassing data from 11 studies, did not reveal a significant association with breast cancer risk.

The ABO gene, found on chromosome 9q34, encodes glycosyltransferases, which allow the transfer of nucleotide sugars to the H antigen, resulting in the development of the ABO blood group antigens [52]. These antigens are found not just on the surface of red blood cells, but also on many other cells and tissues, including the normal breast ductal cells [52]. Glycosyltransferases, crucial for intercellular bonding and signaling across cell membranes, significantly contribute to the advancement and dissemination of cancer [53]. Previous data has shown connections between the A blood group allele genotype and the levels of soluble intercellular adhesion molecule 1, E-selection, and P-selection in circulation [54, 55]. This suggests that blood group A might have an impact on the body’s inflammatory response, possibly contributing to the higher occurrence of breast cancer in women with blood type A.

Numerous studies have explored the connection between ABO blood groups and the development or progression of various human tumors after identifying the association between gastric cancer and blood group A in 1953 [29]. For instance, research has indicated a heightened risk of breast cancer among individuals with blood group A [56]. Similarly, a case-control study revealed an elevated risk of pancreatic cancer in individuals with blood type A [57]. In contrast, another study reported a higher risk of ovarian cancer in women with blood group A compared to those with other blood types [58]. Nevertheless, findings regarding the relationship between blood group A and breast cancer have been inconsistent [35, 44].

A previous meta-analysis [23], including 14 studies revealed no significant correlation between ABO blood types and breast cancer incidence with OR for blood type A, B, and AB. However, within the Caucasian subgroup, individuals with blood type A exhibited a slightly elevated risk of breast cancer compared to other blood groups (OR = 1.066, 95% CI, 1.001–1.134). Nonetheless, no significant associations were detected in other subgroup analyses. Furthermore, no noteworthy link was identified between the Rh factor and breast cancer, which is consistent with our findings.

Our subgroup analysis found a significant association between blood group A and breast cancer risk in non-Asian but not in Asian populations. However, the lack of a significant difference between regions (p^Heterogeneity^ = 0.74) suggests that regional factors do not strongly influence this relationship, possibly due to differences in sample sizes, genetics, or environment.

According to a previous study, blood group A was associated with a higher risk of breast cancer than other blood groups. For instance, patients with blood group A had the highest incidence of breast cancer (45.88%), followed by those with blood group O (31.69%), blood group B (16.16%), and blood group AB (6.27%) [59]. Like our findings, Guleria et al indicated a significant association between blood group A and breast cancer compared to the control group [60]. Also, Mourali et al. found a positive risk association among Tunisian women with rapidly progressive breast cancer with blood group A [61]. In Iceland, a study in 1988 examining bilateral breast cancer risk in familial and sporadic cases found a twofold higher prevalence of type B in familial cases [62]. Other researchers have also reported an increased rate of blood type A in breast cancer patients, suggesting possible masking effects by susceptibility genes or involvement of different etiological mechanisms [28].

Abobaker et al investigated the relationship between ABO blood groups and breast cancer in a primary hospital in Penang, aiming to evaluate the ABO blood group’s utility as a preclinical marker. They identified a significant correlation (30.0%) between blood type A and breast cancer, in contrast to the incidence observed in other blood groups. Nonetheless, statistical significance was not attained for this connection, although blood categories B and O were much less prevalent among cancer patients [25]. Also, Zounie et al reported a significantly increased cancer risk in women under the age of 35 with blood type A [44]. Both blood types A and AB were correlated with a higher frequency of lymph node metastasis (*p* < 0.05) [44]. Blood type B was linked with breast carcinomas that exhibited overexpression of the human epidermal growth factor receptor HER2 (*p* < 0.05) and a heightened risk of cancer onset after the age of 70 (*p* < 0.001). Individuals with blood groups A, B, and AB were more prone to developing aggressive forms of breast carcinoma [44].

Multiple studies have demonstrated an association between the Rh factor and specific forms of breast cancer. Stamatakos et al. identified a greater prevalence of ductal carcinoma in patients who were Rh factor positive, irrespective of their ABO blood group [42]. The closeness of the Rh factor gene to the endothelin-converting enzyme 1 gene on chromosome 1p36.1 renders this relationship feasible. Prior studies emphasized endothelin’s pivotal function in the progression and dissemination of malignant tumors, with its receptors essential for the advancement and invasion of breast cancer cells [63]. These two genes are located in proximity within the 1p36 region [64]. Notwithstanding these findings, our study could not identify a significant association between the Rh factor and breast cancer risk, indicating that more research is required to definitively ascertain this relationship.

We believe that ABO blood groups could aid in breast cancer risk stratification and screening, women with blood type A may require more frequent check-ups, earlier mammograms, or other preventive measures, which might result in early detection.

We believe that ABO blood groups could aid in breast cancer risk stratification and screening, women with blood type A may require more frequent check-ups, earlier mammograms, or other preventive measures, which might result in early detection.

While rare cases of post-surgical ABO phenotype changes have been reported [65, 66], particularly following hematopoietic stem cell transplantation, these are not systemic and do not reflect a true genetic conversion. Since our analysis is based on baseline blood groups, such variations are unlikely to impact the observed association with breast cancer risk.

## MATERIALS AND METHODS

### Protocol

This research was submitted to PROSPERO (CRD42022361407). Our actions are in accordance with the Preferred Reporting Items for Systematic Reviews and Meta-Analyses (PRISMA) recommendations.

### Search strategy

From 1945 to 2022, we searched to verify if any new articles were published during this period suitable for our specific criteria. Still, we did not obtain any new articles. Two reviewers (K.Z.) and (M.A.) conducted a comprehensive examination of articles, and our investigation encompassed the following electronic databases: PubMed (MEDLINE), Scopus, Web of Science (WOS), and Google Scholar. The search parameters and keywords were adjusted for every database. The following keywords were used in this research (Breast) AND ((Tumor) OR (Neoplasm) OR (Cancer) OR (Carcinoma) AND (ABO Blood Group System)) OR (ABO Blood Group System) OR (Blood-Group System, ABO).

### Eligibility criteria

We included studies that followed the following PICOS criteria: included articles available in English, and case-control in design including 100 or more breast cancer cases (not being) has information about ABO blood group type.

We excluded studies if they had one of the following criteria: (1) non-original research (i.e., reviews, commentaries, guidelines, editorials, correspondence, letters to editors, etc.), (2) unavailable full texts, (3) duplicated records or records with overlapping datasets, (4) no clear definition about the tumor (malignant or benign), (5) studies with sample size less than 100 and (6) non-English paper.

### Study selection

The studies were retrieved from the database search, and we used EndNote for duplicate removal, then exported them into an Excel sheet for screening. At first, two authors (K.Z.) and (M.A.) screened the titles and abstracts of the retrieved articles according to our eligibility criteria. Then, relevant studies underwent full-text screenings. Then we conducted manual searches of significant publications and referenced sources mentioned in the included studies. This process was carried out by two sets of two authors who resolved their differences through discussions. Meanwhile, the senior author (R.A.) was consulted when an agreement could not be reached.

### Study quality

We used the Risk of Bias in Non-Randomized Studies of Interventions (ROBINS-I) tool for non-randomized trials [67]. The task was undertaken by two authors (M.A.) and (K.Z.) who addressed their discrepancies through discussions.

### Statistical analysis

We performed statistical analysis using R version 4.2.1(2022-06-23 ucrt) and packages, “meta” and “metafor”. We used the odds ratio (OR) and its 95% CI. A random effects model was applied to pool the data. We used Begg’s funnel plot and Egger’s test for publication bias assessment [68]. Assessment of heterogeneity was assessed by visual inspection of the forest plots and measured by I-square and Chi-square tests. The Chi-square test measures the existence of significant heterogeneity, while the I-square quantifies the magnitude of heterogeneity in the effect size. The heterogeneity of the included studies was assessed using the I² test and corresponding *p*-value, where I² < 50% indicated low heterogeneity, while I² > 50% suggested high one. Statistical significance for the overall effect size was determined by a *p*-value below 0.05. Studies were categorized as ‘Asian’ if they were conducted in countries classified as part of Asia according to the United Nations official statistics.

### Strengths and limitations

To the best of our knowledge, our meta-analysis stands as the most comprehensive exploration of the link between ABO blood type and breast cancer to date. Our rigorous inclusion criteria, which focused on case-control study designs, lend robustness to our findings. The inclusion of diverse ethnic groups in our analysis, coupled with a sizable sample size, enhances the generalizability of our results. Moreover, the consistency across the literature in terms of study type, design, and sample size adds further credibility to our conclusions. However, it is worth noting that our analysis relied on unadjusted odds ratios, potentially overlooking the impact of other variables on the association. Additionally, the scarcity of high-quality studies may have affected the overall strength of evidence available.

While our findings suggest a statistical association between blood type A and breast cancer risk, they do not establish a direct causal relationship. Further research should explore ABO gene polymorphisms, antigen expression patterns, and immune-related pathways to better understand the underlying mechanisms. Incorporating genetic analysis may help clarify the biological relevance of ABO blood groups in breast cancer.

As a meta-analysis, this study is limited by the lack of individual patient data (IPD), preventing multivariate analysis. While meta-regression could assess confounders, the absence of sufficient stratified data in the included studies made this unfeasible. Future research with IPD or adjusted risk estimates is needed to clarify these interactions.

## CONCLUSIONS

This meta-analysis represents the most comprehensive study conducted thus far regarding the association between ABO blood groups and breast cancer risk with a total of 730877 individual (13029, breast cancer cases). The rigorous inclusion criteria employed, which emphasized case-control study designs, large sample sizes, the inclusion of diverse ethnic populations, and the absence of publication bias, lend credibility to the research and enhance the generalizability of the findings.

The results suggest a potential association between blood type A and an increased risk of breast cancer compared to blood type O. However, the association was not statistically significant for blood types B, AB, or Rh.

To better understand the underlying mechanisms and infer causal associations, future research will need to conduct longitudinal studies that follow participants over time and analyze cancer incidence rates across different blood groups. This would help elucidate the biological pathways behind the observed association between ABO blood groups and breast cancer risk, which, in turn, aids in various aspects, including diagnosis, treatment, prevention, and improving the quality of life for individuals affected by breast cancer.

## SUPPLEMENTARY MATERIALS


