# Supplementary material for: Relationship between ABO blood group antigens and Rh factor with breast cancer: A systematic review and meta-analysis
Source: Oncotarget. 2025 May 9;16:311–26. doi: 10.18632/oncotarget.28718 (PMC12068321; doi:10.18632/oncotarget.28718)
Supplement: Supplementary file 1 [file oncotarget-16-28718-s001.pdf]

Relationship between ABO blood group antigens and Rh factor with breast cancer: A systematic review and meta-analysis

SUPPLEMENTARY MATERIALS

Study or Subgroup	Country	95% CI	OR	Weight
**Demographic = Non Asian**				
Zouine (2016)	Morocco	[0.69; 1.07]	0.86	4.4%
Newell (1974)	United States	[0.70; 1.26]	0.94	4.0%
Goldenberg (1958)	United States	[0.88; 1.15]	1.00	4.7%
Akanmu (2002)	Nigeria	[0.64; 1.63]	1.02	3.2%
Aird (1954)	England	[0.92; 1.20]	1.05	4.7%
Anderson (1984)	United States	[1.01; 1.21]	1.10	4.8%
Costantini (1990)	Italy	[0.87; 1.43]	1.12	4.2%
Hartmann (1964)	Norway	[1.02; 1.26]	1.13	4.7%
Ronco (2009)	Uruguay	[0.91; 1.74]	1.26	3.9%
Stamatakos (2009) SPACE	Greece	[0.90; 2.09]	1.37	3.4%
Aly (2014)	Egypt	[1.08; 3.78]	2.02	2.5%
Bothou (2019)	Greece	[2.11; 5.71]	3.47	3.0%
**Total (95% CI)**		**[1.01; 1.26]**	**1.13**	**47.4%**
Heterogeneity: $Tau^2 = 0.0190$; $Chi^2 = 34.56$, df = 11 ($P < 0.01$); $I^2 = 68\%$				
**Demographic = Asian**				
Abbasi (2009)	Iran	[0.18; 0.58]	0.32	2.6%
Mjali (2019)	Iraq	[0.33; 0.59]	0.44	4.0%
Kumar (2015)	India	[0.28; 0.74]	0.46	3.1%
Joudaki (2022)	Iran	[0.42; 1.12]	0.69	3.1%
Alhusseini (2016)	Iraq	[0.41; 1.64]	0.82	2.2%
Al−Ganimi (2020)	Iraq	[0.62; 1.23]	0.87	3.8%
Flavargani (2012)	Iran	[0.57; 1.36]	0.88	3.3%
Ray (1980)	India	[0.84; 1.17]	0.99	4.6%
Sujatha (2016)	India	[0.51; 1.95]	1.00	2.3%
Dogan (2022)	Turkey	[0.80; 1.89]	1.23	3.4%
Urun (2012)	Turkey	[0.97; 1.67]	1.27	4.1%
Mohammad (2016)	Iraq	[0.84; 3.44]	1.70	2.2%
Abobaker (2014)	Malaysia	[1.02; 4.65]	2.17	2.0%
Dixit (2020)	India	[1.25; 4.01]	2.24	2.7%
Bhartiaya (2015)	India	[1.95; 6.13]	3.45	2.7%
Zaki (2013)	Iraq	[2.83; 6.74]	4.36	3.3%
Mehdi (1991)	Iraq	[2.80; 7.11]	4.46	3.2%
**Total (95% CI)**		**[0.86; 1.66]**	**1.19**	**52.6%**
Heterogeneity: $Tau^2 = 0.4139$; $Chi^2 = 175.95$, df = 16 ($P < 0.01$); $I^2 = 91\%$				
**Total (95% CI)**		**[1.03; 1.36]**	**1.18**	**100.0%**
Heterogeneity: $Tau^2 = 0.1050$; $Chi^2 = 210.70$, df = 28 ($P < 0.01$); $I^2 = 87\%$				
Test for subgroup differences: $Chi^2 = 0.11$, df = 1 ($P = 0.74$)				

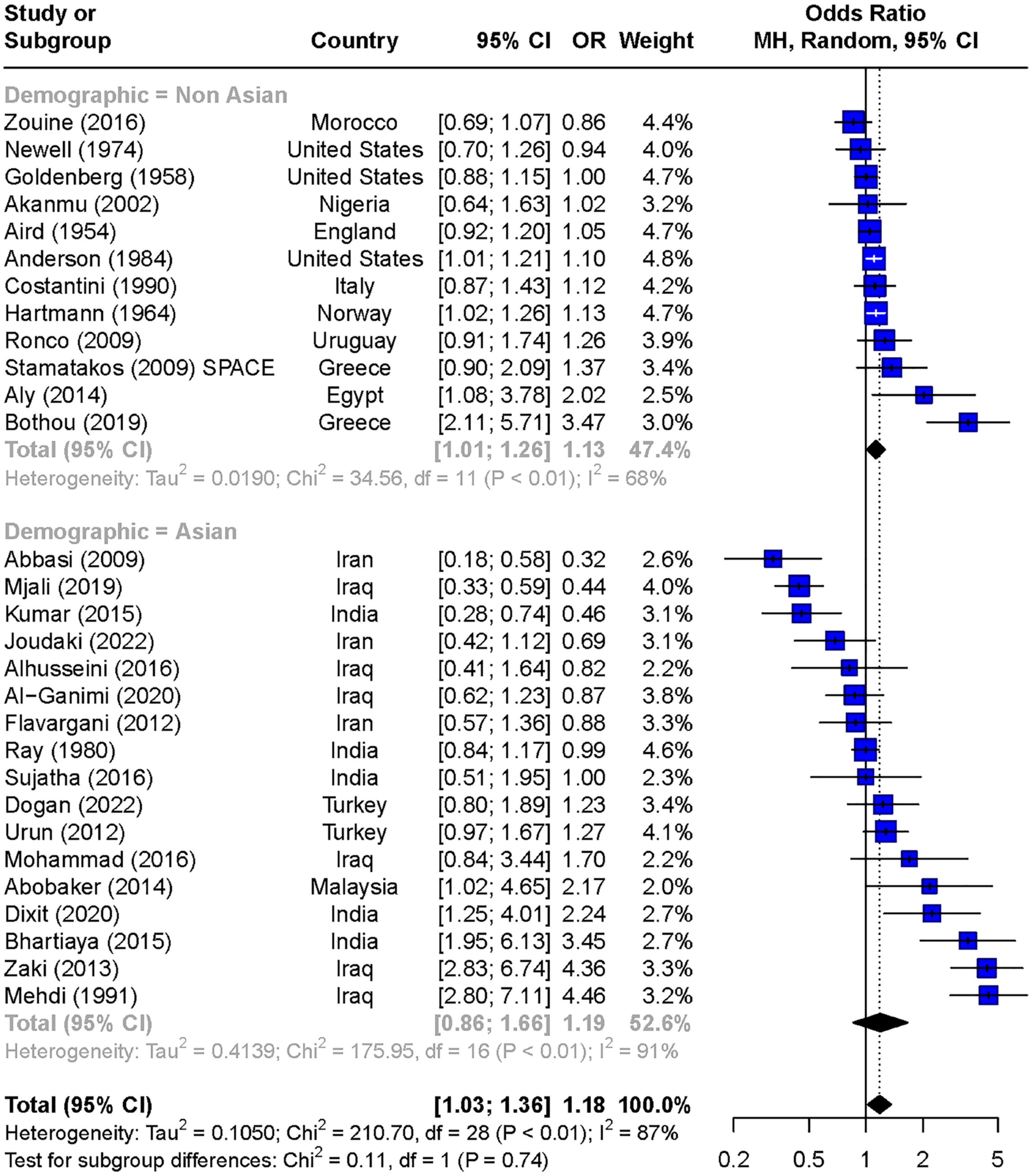

Supplementary Figure 1: Forest plot of the odds ratios for breast cancer risk comparing blood group A to blood group O, with a subgroup analysis for Asian and non-Asian patients.

Supplementary Table 1: Egger test values

Group	intercept	95% CI	t value	*p*-value
A/O	0.855	–0.84–2.55	0.988	0.331
B/O	–1.043	–2.27–0.18	–1.668	0.106
AB/O	0.589	–0.66–1.84	0.921	0.364
Rh+/Rh-	1.197	–1.34–3.73	0.925	0.378
